# Lower Heart Rate Variability is Associated with High Disease Activity, Functional Disability and Inflammation in Rheumatoid Arthritis: A Cross-Sectional Study

**DOI:** 10.31138/mjr.290524.lhv

**Published:** 2025-01-30

**Authors:** Suchitra Lakshmi Goda, Madhumitha Haridoss, Krishnamurthy Venkataraman, Bhavani Shankara Bagepally

**Affiliations:** 1 Health Technology Assessment Resource Centre, ICMR-National Institute of Epidemiology, Chennai, India;; 2 Division of Medical Research, Faculty of Medicine and Health Sciences, SRM Medical College, Hospital and Research Centre, SRM Institute of Science and Technology, Kattankulathur, Chennai, India;; 3 Chennai Meenakshi Multispecialty Hospital, Chennai, India

**Keywords:** rheumatoid arthritis, ECG, HRV, DAS-28, HAQ capacity

## Abstract

**Introduction:**

Rheumatoid arthritis (RA) is a chronic autoimmune disorder characterised by persistent inflammation that negatively impacts cardiovascular health, often leading to cardiac autonomic neuropathy. Heart Rate Variability (HRV), a non-invasive measure, reveals compromised autonomic regulation in RA, as evidenced by reduced HRV and increased sympathetic control in RA. Despite existing evidence, the relationship between HRV and RA remains inconclusive, prompting this study to examine HRV in RA disease activity.

**Methods:**

Study involved 320 individuals with RA, aged ≥18 years, attending an outpatient clinic at a tertiary care multispecialty hospital in south India. Sociodemographic, clinical, and laboratory data, and HRV parameters, were obtained. RA disease-specific parameters such as Health-Related Quality of Life, Disease activity and Functional status were assessed using EuroQol-5D, Disease Activity Score-28 (DAS-28) and Health Assessment Questionnaire (HAQ) respectively. Spearman’s correlation and linear regression analyses were performed to examine the association between HRV and RA disease-specific parameters. A statistical significance level of p<0.05 was considered.

**Result:**

We observed a significant negative correlation between HRV parameters and RA disease-specific parameters such as DAS-28, HAQ and Erythrocyte sedimentation rate. The linear regression analysis indicated that a lower Standard Deviation rate Beats Per Minute was linked to higher functional ability scores according to the HAQ Scale.

**Conclusion:**

Reduced HRV in individuals with RA is linked to higher disease activity, functional disability and inflammation. This underscores the significance of HRV as a potential tool for assessing cardiovascular autonomic function in RA patients, with implications for their management and prognosis.

## INTRODUCTION

Rheumatoid arthritis (RA) is a chronic, autoimmune disorder characterised by a progressive course, culminating in substantial pain, joint degradation, and declining function.^1^ The persistent inflammatory milieu in RA exerts a considerable toll on cardiovascular health.^2^ Cardiac autonomic neuropathy is documented as one of the most frequent side effects of RA, bearing significant mortality risks attributed to arrhythmias and myocardial infarctions.^3,4^ Earlier investigations revealed a substantial proportion of RA patients, ranging from 61% to 75%, exhibiting Cardiovascular autonomic dysfunction,^5^ marked by subdued parasympathetic response coupled with an augmented sympathetic nervous system. The existing contradictory evidence on sympathetic and parasympathetic activities in RA underscores the necessity for newer studies aimed at a comprehensive grasp of these dynamics.^6^

Heart Rate Variability (HRV) emerges as a non-invasive approach for the early detection of autonomic cardiac impairment.^7^ It offers a superior quantitative and qualitative assessment of sympathovagal regulation over cardiovascular function.^8^ Lower HRV often signifies compromised autonomic nervous system regulation and is correlated with an elevated susceptibility to cardiovascular events.^9^ An increase in sympathetic control of the heart rate has been observed in RA patients,^10^ and a number of studies have consistently reported decreased HRV in RA patients.^10–13^ Nunan et al. and Malik et al. highlights key HRV parameters for assessing autonomic function. Hence, in this manuscript, we have mentioned only few clinically significant HRV parameters. Time-domain measures like SDNN and RMSSD reflect overall and short-term HRV, with lower values of those parameters linked to cardiovascular risk, stress, and anxiety.^17^ Frequency-domain measures, including LF, HF, and the LF/HF ratio, provide insight into autonomic balance, with higher LF/HF ratios indicating increased sympathetic dominance and cardiovascular risk.^18^ Nonlinear measures, such as Poincaré Plot Indices (SD1 and SD2), offer additional insights into short-and long-term HRV, with changes linked to various clinical conditions.^19^

Prior investigations have also established a connection between increased mortality among RA patients, elevated heart rate, and reduced HRV. ^8^ Despite evidence suggesting a link between diminished HRV and RA, the results have been inconclusive.^3^ Additionally, there is a shortage of studies examining the link between HRV and disease activity in RA. In line with this context, the present study evaluates HRV in RA patients exhibiting varying degrees of disease activity.

## METHODS

We reported this manuscript according to the STROBE (Strengthening the Reporting of Observational Studies in Epidemiology) checklist.^14^

### Study Design and Participant Selection

This study was conducted at a private multispecialty hospital in Chennai, Tamil Nadu, India, using a cross-sectional descriptive design. The study focused on patients with RA who visited the outpatient clinic. A total of 320 RA patients who met the inclusion criteria were included in the study. The recruitment of participants took place between April and October 2022.

### Eligibility Criteria

Patients who were 18 years of age or older and had been diagnosed with RA according to the 2010 RA classification criteria (Al et aha, 2010) were considered eligible. Additionally, patients were required to have had at least one follow-up visit after their diagnosis. Individuals with other rheumatic or autoimmune diseases were not included in the study.

#### Exclusion Criteria

We collected data exclusively from a rheumatology clinic, only RA patients were included in the study. Patients with other conditions, including malignancy, neurological disorders, and pregnancy, were excluded.

### Sample size determination and participation selection strategy

The sample size for this study was estimated based on a remission rate of 30% among RA patients, as reported by Hoshi et al. (2016),^15^ with a relative precision of 15% (i.e., 4.5% absolute precision). Given a total population of 1000 registered RA patients in the hospital and a 95% confidence level, the initial sample size was calculated to be 286. To account for an anticipated 10% non-response rate, the final sample size was adjusted to 320 participants. This calculation was performed using OpenEpi, Version 3, to ensure sufficient statistical power and precision in estimating the remission rate among RA patients.

The final sample size of 320 was determined based on the prevalence of different RA severities, considering a 15% relative precision, 95% confidence interval, 10% non-response rate, and a design effect of 1. Systematic sampling was employed to select participants, with every third patient meeting the inclusion criteria being recruited after providing written informed consent. The study included RA patients attending the outpatient clinic of a multispecialty hospital. Patients were selected in a systematic manner based on their order of arrival or registration; the first patient would be included, while the second and third were skipped, the fourth included, and so on. This systematic selection process ensured a representative sample without consecutive patient selection.

### Data Collection

Baseline characteristics, including sociodemographic, clinical, and laboratory data, were gathered through personal interviews with the participants and referring to their medical records. A standardised paper-based structured questionnaire, developed specific to the study, was used for data collection. The data collectors received comprehensive training on participant recruitment, data collection, and data entry. The collected data were directly entered into MS Excel, and to ensure data accuracy, a second person cross-checked the entered data. Additionally, numerous blood parameters were collected during the study. However, among the 320 participants, CRP measurements were unavailable for 74.4% (238 participants) of the individuals.

Apart from this, all 320 participants had a questionnaire-based interview about the health-related Quality of Life, which was assessed using Euro QoL-5 Dimension (EQ-5D) scale. The functional assessment of the RA patients was assessed using Health Assessment Questionnaire (HAQ) and the disease Severity was measured using the Disease Activity Score (DAS-28) scale. EQ-5D is a frequently utilised tool for gauging patient-reported outcomes (PROs) related to health. It is also recommended by the National Institute for Health and Care Excellence (NICE) as a preferred approach for evaluating health state utilities in the context of health technology assessment (HTA).^16^

### Ethics and consent

The study adhered to the ethical guidelines outlined in the Declaration of Helsinki and the ICMR guidelines. The research protocol obtained approval from the ethics committees of both ICMR-National Institute of Epidemiology (ICMR-NIE) (NIE/IHEC/202101-01) and CMMH (CMMHEC/21/09). Prior to participation, all study participants provided written informed consent, which was witnessed by an authorised individual. A record of the signed informed consent form is retained in the study documentation.

### Recording of HRV

As part of the study, participants underwent HRV measurement. Among 320 participants, six participants declined to participate in the HRV recording. Hence, 314 samples were considered for the analysis. Each participant was assigned a referral ID or PID (Personal Identification Number) for the HRV measurement. The HRV recording took place during the participant’s routine check-up at the hospital. After explaining the test procedure, the participant was instructed to lie comfortably in a noise-free environment. Skin preparation was conducted using a Spirit swab. The HRV recording utilised an ECG type of instrument named AD instruments power lab 15T (Model no. ML 4818. S/N: T15-3027) and involved the application of conduction gel to five specific areas where the machine leads were placed. Only five leads were used for collecting the HRV data, with the leads connected to the following areas: 1. Right wrist, 2. Left wrist, 3. Right ankle, 4. Left ankle, and 5. An additional earth lead was placed at the right wrist. Before recording, the connection between the AD instrument and the laptop containing the AD instruments Lab chart Pro software (version- v8.1.7 04-08-2017copyright 1994–2017 AD instruments) was verified. The lab chart page was opened, and the ECG recording was performed for 10 minutes. A minimum of a 5-minute recording segment that was free of noise and artefacts was selected for analysis.

### Measurement of HRV

The recording of each participant was saved, and initially, the presence of any ectopic beats was verified. Out of 17 HRV parameters only 9 parameters are considered for the analysis due to its clinical significance. Measurement of the HRV parameters, including SDNN, RMSSD, and NN50, in time domain analysis and LF, HF, LF/HF, in frequency domain analysis was done. The following HRV parameters were derived from the ECG recordings: the standard deviation of all RR intervals in ms (SDRR or SDNN), Standard deviation of Beats per minute in ms (SD rate in BPM), the square root of the mean of the sum of the squares of differences between adjacent RR intervals in ms (RMSSD), percentage of NN50 count of all RR intervals, also known as pNN50 (pRRX%), Poincare plot standard deviation perpendicular the line of identity (SD^1^) in ms, Poincare plot standard deviation along the line of identity (SD^2^) in ms, Absolute power of low-frequency band (LF Power in ms²), Absolute power of the high-frequency band (HF power in ms²), and LF/HF ratio (ratio of LF power to HF power).

### Statistical analysis

The summary or clinical characteristics and HRV parameters of the patients were presented as the mean and standard deviation (SD) for continuous data, while categorical data were expressed as numbers and percentages. The HRV parameters were further categorised based on Quality of life (measured by EQ5D), disease activity (measured by DAS-28) and functional status (measured by HAQ). Spearmen correlation and linear regression analyses were conducted to identify independent factors associated with HRV and EQ-5D, HAQ and DAS-28 scores. A p-value less than 0.05 was considered statistically significant. All data analyses were performed using SPSS version 25 (IBM Corp, 2017).

## RESULTS

### Clinical characteristics

This study examined 320 RA individuals whose mean age was 55.5 ± 12.3 years, among which 87.8% of them were female. The characteristics of the study participants are presented in **Table 1**.

**Table 1.** Clinical characteristics of RA patients.

**Clinical characteristics**	**Mean ± SD (n=320)**
Sample Size	320
Age (yrs.)	55.5 ±12.3
Female	87.8%
BMI	27.3±5.6
BP-Systolic (mm/hg)	122.3±16.3
BP-Diastolic (mm/hg)	78.5±8.3
ESR (mm/hour)	43.2±23.1
Disease duration (years)	8.6±7.5
Rheumatoid Factor Positive (%)	238 (74.4%)
Heart Rate	665.5
DAS -28 Score	4.71

BMI: body mass index; BP: blood pressure; ESR: erythrocyte sedimentation rate; hg: mercury; IU: international units; ml: millilitre; mm: millimetre.

### List of self-reported Ailments, Personal Habits, Types of medication

**Table 2** contains details on several sociodemographic details. Other ailments, such as peptic ulcers, renal disease, and various inflammatory conditions, include cases with one or two affected individuals.

**Table 2.** List of self-reported ailments, personal habits, and types of medication.

**Variables**	**n (%)**
**Self-reported Ailments**	
Hypertension	101 (31.5)
Diabetes Mellitus	84 (26.2)
Cardiovascular Disease	5 (1.5)
Asthma	11 (3.4)
Hyperlipidaemia	16 (5)
**Personal Habits**	
Smoking	9 (2.8)
Alcohol	8 (2.5)
**Types of medication**	
Deflazacort	77 (24)
Prednisolone	(65)
Methotrexate	287 (89.6)
HCQ	260 (81.2)
Sulfasalazine	26 (8.1)
Leflunomide	35 (16.5)

HCQ: hydroxychloroquine.

### HRV parameters

HRV was assessed in all the study participants. Among the 320 participants, the ECG was not recorded for six individuals. Hence, only 314 individuals were considered for analysis. The description of 9 HRV parameters is presented in **Table 3**. HRV parameters showed low variability in RR intervals, as indicated by the standard deviation (SDRR) of 32.6 ms.

**Table 3.** HRV parameters.

**HRV parameters**	**(n=314) Median ± IQR**	**Normal Range**
SD rate BPM	3±2.2	60–100 BPM
SDRR ms or SDNN	32.6±31.5	50–150 ms
RMSSD ms	27.2±42.8	20–50 ms
pRRx%	1.1±8.1	10–30%
SD 1 ms^2^	19.2±30.3	20–50 ms
SD 2 ms^2^	39.6±32.1	100–150 ms
LF power ms^2^	186.8±426.3	0.04–0.15 Hz
HF power ms^2^	261.7±928.9	0.04–0.15 Hz
LF/HF	0.6±1.02	0.5 and 2.0

SDRR or SDNN: the standard deviation of all RR intervals in ms; SD Rate in BPM: Standard deviation of Beats per minute in ms; RMSSD: the square root of the mean of the sum of the squares of differences between adjacent RR intervals in ms; pRRX%: percentage of NN50 count of all RR intervals (also known as pNN50); SD 1 in ms: Poincare plot standard deviation perpendicular to the line of identity; SD 2 in ms: Poincare plot standard deviation along the line of identity; LF power in ms^2^: Absolute power of the low-frequency band; HF power in ms²: Absolute power of the high-frequency band; LF/HF ratio: ratio of LF power to HF power; ms: milliseconds.

### Correlation

**Table 4** represents the Spearman correlation analysis, which revealed a statistically significant negative correlation between various HRV parameters, including SDRR, SD rate BPM, RMSSD, SD ms^2^, LF power ms^2^, and HF power ms^2^ with disease-specific parameters such as HAQ, DAS-28, and ESR. SD rate BPM exhibited a negative correlation with HAQ and DAS-28, while not significantly correlated with ESR. Average RR, Mean RR and average rate BPM showed negative correlations only with ESR. None of the HRV parameters demonstrated significant correlations with EQ-5D, disease duration and disease specific factors except HAQ.

**Table 4.** Spearman correlation with HRV, disease-specific parameters, and inflammatory factor.

**HRV parameters**	**HAQ**	**DAS-28**	**EQ5D**	**DD (based on years)**	**ESR**
SD Rate BPM	−.158^**^	−.171^**^	.086	.068	−.105
SDRR	−0.135^*^	−0.166^**^	0.098	0.075	−0.153^**^
RMSSD	−0.084^*^	−0.143^*^	0.065	0.099	−0.122^*^
pRRX%	−0.139	−0.159^**^	0.057	−0.013	−0.179^**^
SD1 ms 2	−0.084^*^	−0.143^*^	0.065	0.099	−0.122^*^
SD 2 ms2	−0.160^**^	−0.176^**^	0.117^*^	0.061	−0.170^**^
LF power ms2	−0.155^**^	−0.185^*^	0.097	0.023	−0.190^**^
HF power ms2	−0.114^*^	−0.131^*^	0.047	0.054	−0.139^*^
LF/HF	−0.008	−0.045	0.054	−0.083	−0.007

* Correlation is significant at the 0.05 level (2-tailed)

** Correlation is significant at the 0.01 level (2-tailed)

EQ-5D: EuroQoL-5 Dimension scale; DAS-28: Disease Activity Score; HAQ: Heath Assessment Questionnaire; DD: disease duration; ESR: erythrocyte sedimentation rate.

### Linear regression analysis

A linear regression analysis was conducted to investigate further the relationship between HAQ Score and SD rate BPM. SD rate BPM was the only predictor variable entered in the model. The model summary (**Table 5**) indicated a weak overall fit (R = 0.138, R Square = 0.019, Adjusted R Square = 0.016). The standard error of the estimate was 0.650536. The change statistics demonstrated that the addition of SD rate BPM significantly improved the model fit [R Square Change = 0.019, F (1, 312) = 6.036, p = 0.015]. The Durbin-Watson statistic was 1.785, indicating no significant autocorrelation. The ANOVA results indicated that the regression model was statistically significant [F (1, 312) = 6.036, p = 0.015], suggesting that SD rate BPM had a significant impact on HAQ Score. The coefficient table revealed that SD rate BPM had a significant negative association with HAQ Score [B = −0.033, SE = 0.013, β = −0.138, t = −2.457, p = 0.015]. This indicated that for every one-unit decrease in SD rate BPM, HAQ Score increased by 0.033, after controlling for other variables. The residual statistics showed that the predicted values of the HAQ Score ranged from 0.42723 to 1.17643, with a mean of 1.08320. The residuals had a mean of zero (Mean = 0.000000) and a standard deviation of 0.649496. Overall, the findings from the linear regression analysis supported the significant positive relationship between SD rate BPM and HAQ Score, suggesting that lower SD rate BPM was associated with higher functional ability scores according to the HAQ Scale.

**Table 5.** Model summary of linear regression.

**Model**	**R**	**R Square**	**Adjusted R Square**	**Std. Error of the Estimate**	**Change Statistics**					**Durbin-Watson**
					**R Square Change**	**F Change**	**df1**	**df2**	**Sig. F Change**	
1	.138^a^	.019	.016	.650536	.019	6.036	1	312	.015	1.785

a Predictors: (Constant), SD rate BPM.

b Dependent Variable: HAQ Score.

## DISCUSSION

The study assessed the HRV in individuals with RA exhibiting varying levels of disease activity. We observed a significant negative correlation between HRV parameters and RA disease-specific parameters such as DAS-28, HAQ and ESR. Our results imply that a diminished HRV serves as an indicator of high disease activity, as determined by the DAS28 score, and increased functional disability, as measured by the HAQ, in individuals with RA. Furthermore, the outcomes of the linear regression analysis supported the notion that an elevated SD rate expressed in beats per minute (BPM) is linked with improved functional status among individuals with RA. These results collectively contribute to understanding the relationship between HRV, disease activity, and functional status in individuals with RA. DAS-28 and HAQ scores have been used as primary or secondary outcomes in many RA trials for evaluating drug response. Our findings indicate reduced HRV in RA individuals with high disease activity, suggesting that HRV might serve as a predictive tool for assessing drug response in RA patients. A proof-of-concept study with a limited sample of RA patients has shown that individuals with poor HRV profiles may exhibit an inadequate response to anti-tumor necrosis factor-alpha agents.^18^ Additionally, an economic exploratory analysis^19^ suggests that HRV testing and ANS optimisation prior to initiating biologics in refractory RA patients could be cost-effective. HRV signals have also been associated with personalised drug responses in various medical conditions, including major depressive disorder, epilepsy, chronic pain, and hypertension. However, the relationships between HRV signals and personalised drug responses across different diseases and patient populations remain complex and not fully understood.

Prior investigations have compared HRV between healthy individuals and those with RA, revealing reduced HRV in the latter group. Our study took a different approach by examining associations between HRV and RA-specific disease-related parameters. Our findings suggest that RA patients with high disease activity and severe functional disability may have a higher risk of cardiovascular events due to diminished HRV. In general, a reduced HRV indicates an abnormal autonomic nervous system and is associated with a higher risk of cardiovascular events.^11^ An imbalance between HRV and sympathetic-vagal activity, with an increased sympathetic or reduced vagal activity, has been identified as a significant and separate predictor of mortality following a heart attack. It also has prognostic value in patients with heart failure and diabetic neuropathy. Thus, monitoring HRV in RA patients may provide valuable insights into their autonomic function and potential cardiovascular risks, ultimately aiding in their clinical management and improving their overall health outcomes.

There are a few limitations in our study. We have not used healthy controls. Therefore, a comparative HRV profile between healthy and RA individuals could not be drawn. The sample size of our study may be insufficient to study the potential of utilising HRV to predict response to various DMARDs in RA patients. Therefore, definitive conclusions could not be reached in this regard. The cross-sectional design of the study limits our ability to determine if changes in HRV cause changes in disease activity or functional status. Long-term studies are needed to understand the timing and cause-and-effect relationships. Data was collected from a single rheumatology clinic, which may limit the generalisability of the findings to broader RA patient populations across different geographic regions and healthcare settings. Due to the presence of comorbidities, among the RA patients which could influence both the HRV and RA disease activities it was harder to determine the true relationship between HRV and RA patients.

## CONCLUSION

This study demonstrates that reduced HRV is associated with higher disease activity and severe functional disability in RA patients. Existing evidence and our findings collectively underscore the potential utility of HRV as a predictive marker for drug response in RA and other medical conditions. Further research and exploration are warranted to elucidate the intricate relationships between HRV signals and individualised drug responses, thereby advancing our understanding and application of this promising avenue in RA treatment. Our study also highlights the importance of HRV as a potential tool to assess cardiovascular risk in RA patients and may have implications for their management and prognosis.

## AUTHORS CONTRIBUTION

Suchitra Lakshmi: Data curation, Formal analysis, Original draft.

Madhumitha Haridoss: Conceptualisation, Formal analysis, review & editing.

Krishnamurthy Venkataraman: Clinical samples, review and editing.

Bagepally BS: Conceptualisation, Data curation, Formal analysis, review & editing.

## CONFLICT OF INTEREST

The authors declare no conflict of interest.

## FUNDING

We received no specific funding for this work. However, the Dept. of Health Research Govt. of India funds the Health Technology Assessment resource center ICMR-NIE. Funders had no role in the conceptualisation, conduction, and manuscript preparation.

## DATA AVAILABILITY

Data cannot be shared publicly due to ethical reasons as it contains individual patient details (confidential data). Data are available from the HTARC, ICMR NIE (contact via Dr. Bhavani Shankar bagepally.bs@gov.in) for researchers who meet the criteria for access to confidential data.
